# Mitochondrial DNA copy number in autism spectrum disorder and attention deficit hyperactivity disorder: a systematic review and meta-analysis

**DOI:** 10.3389/fpsyt.2023.1196035

**Published:** 2023-07-05

**Authors:** Ghada Al-Kafaji, Haitham Ali Jahrami, Materah Salem Alwehaidah, Yasmeen Alshammari, Mariwan Husni

**Affiliations:** ^1^Department of Molecular Medicine and Al-Jawhara Centre for Molecular Medicine, Genetics, and Inherited Disorders, College of Medicine and Medical Sciences, Arabian Gulf University, Manama, Bahrain; ^2^Department of Psychiatry, College of Medicine and Medical Sciences, Arabian Gulf University, Manama, Bahrain; ^3^Government Hospital, Manama, Bahrain; ^4^Department of Medical Laboratory, Faculty of Allied Health, Kuwait University, Kuwait City, Kuwait; ^5^Faculty of Medicine, Dar Al Uloom University, Riyadh, Saudi Arabia; ^6^Department of Psychiatry, Northern Ontarion School of Medicine University, Thunder Bay, ON, Canada

**Keywords:** neurodevelopmental disorders, ASD, ADHD, mtDNA copy number, mitochondrial dysfunction

## Abstract

**Background:**

Several reports suggest that altered mitochondrial DNA copy number (mtDNA-cn), a common biomarker for aberrant mitochondrial function, is implicated in autism spectrum disorder (ASD) and attention deficit hyperactivity disorder (ADHD), but the results are still elusive.

**Methods:**

A meta-analysis was performed to summarize the current indication and to provide a more precise assessment of the mtDNA-cn in ASD and ADHD. A search in the MEDLINE-PubMed, Scopus, and EMBASE databases was done to identify related studies up to the end of February 2023. The meta-analysis was conducted according to recommendations of the Cochrane Handbook of Systematic Reviews.

**Results:**

Fourteen studies involving 666 cases with ASD and ADHD and 585 controls were collected and judged relevant for the systematic review and meta-analysis. The pooled results by a random effects meta-analysis was reported as a geometric mean of the estimated average response ratio and 95% confidence interval. Overall analysis of studies reported differences in mtDNA-cn in blood samples (*k* = 10) and non-blood samples (brain tissues and oral samples; *k* = 4) suggested significantly higher mtDNA-cn in patients compared to controls (*p* = 0.0275). Sub-analysis by stratifying studies based on tissue type, showed no significant increase in mtDNA-cn in blood samples among patients and controls (*p* = 0.284). Conversely, higher mtDNA-cn was observed in non-blood samples in patients than in controls (*p* = 0.0122). Further stratified analysis based on blood-cell compositions as potential confounds showed no significant difference in mtDNA-cn in peripheral blood samples of patients comparted to controls (*p* = 0.074). In addition, stratified analysis of aged-matched ASD and ADHD patients and controls revealed no significant difference in mtDNA-cn in blood samples between patients and controls (*p* = 0.214), whereas a significant increase in mtDNA-cn was observed in non-blood samples between patients and controls (*p* < 0.001). Finally, when the mtDNA-cn was analyzed in blood samples of aged-matched patients with ASD (peripheral blood, leukocytes, and PBMCs) or ADHD (peripheral blood), no significant difference in mtDNA-cn was observed between ASD patients and controls (*p* = 0.385), while a significant increase in mtDNA-cn was found between ADHD patients and controls (*p* = 0.033).

**Conclusion:**

In this first meta-analysis of the evaluation of mtDNA-cn in ASD/ADHD, our results show elevated mtDNA-cn in ASD and ADHD, further emphasizing the implication of mitochondrial dysfunction in neurodevelopmental disorders. However, our results indicate that the mtDNA-cn in blood is not reflected in other tissues in ASD/ADHD, and the true relationship between blood-derived mtDNA-cn and ASD/ADHD remains to be defined in future studies. The importance of blood-cell compositions as confounders of blood-based mtDNA-cn measurement and the advantages of salivary mtDNA-cn should be considered in future studies. Moreover, the potential of mtDNA-cn as a biomarker for mitochondrial malfunction in neurodevelopmental disorders deserves further investigations.

## 1. Introduction

Neurodevelopmental disorders represent a group of heterogeneous conditions that affect the brain function and neurological development, causing impairments in cognition, communication, behavior, and motor functioning (1, 2). The symptoms and behaviors of neurodevelopmental disorders usually appear and diagnose during childhood, but typically persist across a lifespan (3). Individuals with these disorders often experience difficulties in socialization and a reduction in self-esteem and interpersonal skills (2). In the framework of the fifth edition of the Diagnostic and Statistical Manual of Mental Disorders (DSM-5), conditions fall under the umbrella of neurodevelopmental disorders include intellectual developmental disorders, communication disorders, autism spectrum disorder (ASD), attention deficit hyperactivity disorder (ADHD), specific learning disorder, and motor disorders (1). Among these conditions, ASD and ADHD are the two most common neurodevelopmental disorders observed in childhood. ASD is characterized by delayed or abnormal language development, deficits in social interaction, repetitive behaviors and restricted interest, whereas ADHD is characterized by severe deficits in attention with or without hyperactivity and impulsivity (1, 4). Despite the differences in the core symptoms and diagnostic criteria of ASD and ADHD, these two disorders frequently co-occur (5). In this context, a significant proportion (30–80%) of individuals with ASD present with ADHD, and ASD occurs in about 20–50% of ADHD individuals (6). The phenotypic overlap between ASD and ADHD has been explained, at least partly, by shared genetic factors (7–9). As with most complex diseases, other factors besides genetics such as environmental, infectious, and traumatic factors among others contribute to neurodevelopmental disorders (10). Studies have also suggested that common dysfunctional pathways involving mitochondrial activities could account for multiple clinical signs in neurodevelopmental disorders (11–13). Mitochondria produce most of the cellular energy through oxidative phosphorylation, and are also an important source reactive oxygen species (ROS). Beside energy production, mitochondria play a vital role in calcium homeostasis, innate immune, inflammatory responses, red-ox balance and apoptosis (14). Mitochondria have their own genome (mtDNA), which encodes 37 essential genes for proper mitochondrial and cellular functions (15). Each human cell contains multiple copies (about 1,000–10,000) of mtDNA (16). The mtDNA-cn reflects the abundance of mitochondria within a cell and can vary according to the cell’s energy requirement (16, 17). The mtDNA-cn may also differ according to age and general age-related decline in mtDNA-cn was observed across tissues and species (18). Moreover, a study by Chu et al. (19) showed that parental mtDNA-cn was significantly lower than that of their children. Several factors make the mtDNA particularly vulnerable to oxidative stress and other sources of genotoxic damage such as its close proximity to the site of ETC-mediated *ROS* production, lack of protective histones and limited DNA repair capacity. mtDNA oxidative damage can lead to mutations or changes in mtDNA-cn, which may finally lead to mitochondrial dysfunction with more ROS production (20–22). Changes in the mtDNA-cn could alter the expression of mitochondrial genes and cause abnormalities in mitochondrial function and energy production. The mtDNA-cn can be measured by real-time PCR as the ratio of mtDNA copies per nucleated cell in different cell types such as blood, urine, saliva, and tissue biopsies. In previous studies, altered mtDNA-cn was linked with various human diseases such as cancer, neurodegenerative diseases, and psychiatry conditions (23–25) and was suggested as a potential biomarker of these disease. Recent evidence shows that quantitative changes in mtDNA in different tissues are associated with the pathogenesis of neurodevelopmental disorders. For example, increased mtDNA-cn in the peripheral blood was reported in patients with ASD and correlated with neurological manifestations and communication (26). Conversely, a decrease in mtDNA-cn in leukocytes (27) and peripheral blood mononuclear cells was observed in ASD patients (28). On the other hand, no significant difference in mtDNA-cn in temporal lobe was found between ASD patients and controls, suggesting that mitochondrial abnormalities in ASD may be triggered by mechanisms other than mitochondrial biogenesis (29). In addition, higher mtDNA-cn was reported in the peripheral blood of patients with ADHD (30), and remained elevated after treatment (31). These studies imply a complicated role of altered mtDNA-cn in blood and other tissues in ASD and ADHD. Since the involvement of mtDNA-cn in these disorders remains ambiguous, we performed a meta-analysis to summarize the current evidence and to provide more conclusive results of the relationship between mtDNA-cn and ASD/ADHD. We also analyzed changes in mtDNA-cn in different biological samples, and in aged-matched patients and controls.

## 2. Methods

### 2.1. Search strategy

A search for relevant studies that investigated mtDNA-cn in neurodevelopmental disorders was performed using the MEDLINE-PubMed, Scopus, and EMBASE databases up to the end of February 2023. Published studies as primary research were included in the current review and meta-analysis. The following keywords were used for searching: “mitochondrial DNA,” “copy number,” “mtDNA,” “ASD,” “ADHD,” “learning disability,” “intellectual disability,” and “communication disorders.” Other alternative spelling of “copy number” such as “content” was also used. Only those publications in English were retained and the references of related studies were reviewed manually.

### 2.2. Study selection

Studies that met the following criteria were considered eligible for this meta-analysis: 1) studies performed on humans, 2) evaluation of mtDNA-cn in a case–control or a cohort study, 3) mtDNA-cn was measured by previously described methods such as quantitative real time PCR (qPCR), and 4) data were presented as mean mtDNA-cn and its standard or could be transformed from the available data. Publications were excluded if they were: 1) reference abstracts, review articles, editorials or case reports, 2) animal studies, 3) studies with unavailable data for extraction or estimation of mtDNA-cn, and 4) studies focused only on mitochondrial biochemical markers or mtDNA variants/haplogroups.

### 2.3. Data collection

Two investigators first independently assessed the eligibility of studies at the level of title and abstract, with a third reviewer determining the divergences. The full text of suitable articles was retrieved after the agreement was reached by consensus. Discrepancies were discussed and resolved among the investigators and a third reviewer. Relevant data regarding study characteristics were collected on an EXCEL sheet. All identified references and list of publications were transferred to ENDNOTE X 8.2 for Windows.

### 2.4. Assessment of study quality and risk of bias

The Newcastle–Ottawa quality assessment scale (NOS) was used for assessment of study quality (32). Higher quality studies were those with an NOS score > 6. Studies with NOS scores of 8 or 7 points were considered to have a minimal risk of bias and studies with an NOS score of 6 were considered to have a medium risk of bias. Studies with an NOS score of ≤5 were considered to have a high risk of bias. After quality assessment was completed, conflicting judgments were discussed until a consensus was reached.

In the present meta-analysis, the quality assessment of included studies was conducted to evaluate the reliability and validity of the findings (33). We used traffic light plot and summary plot which are the two graphical tools commonly used to present the results of quality assessment in meta-analyses (34). The risk of bias in each included study is represented visually in the traffic light plot (34). Green, yellow, and red traffic light colors are commonly used to denote the degree of bias risk in each study. Green denotes a low likelihood of prejudice, yellow a moderate likelihood of bias, and red a high likelihood of bias. A square or circle is used to symbolize each study, and the size of square or circle reflects the importance of the study in the meta-analysis (34). This method allows quick determination of studies with a high risk of bias, which may have a stronger impact on the overall findings of the meta-analysis (34).

The summary plot is bar plot displaying the percentage of studies within each topic that meet a certain risk of bias judgment (34).

### 2.5. Data analysis

The analysis was performed using mtDNA-cn as the primary outcome. Statistics were presented after a transformation was applied to the raw data. The geometric mean and its 95% confidence interval (95% CI) were used to perform a random-effects meta-analysis. The estimation of heterogeneity (tau^2^) was done using the restricted maximum-likelihood estimator (35). In addition, the Q-test for heterogeneity (36) and the I^2^ statistics were reported. When heterogeneity (tau^2^ > 0) was detected regardless of the Q-test results, a prediction interval for the true outcomes was testified. Studentized residuals and Cook’s distances were used to examine the outliers and/or influential of studies. Studies were considered potentially outliers when a studentized residual was more than 100 x percentile of a standard normal distribution, a Bonferroni correction with two-sided alpha = 0.05 for included studies in the meta-analysis. Studies were considered to be influential when a Cook’s distance was more than the median plus six times the interquartile range of the Cook’s distances. Check for funnel plot asymmetry was done using the rank correlation test and regression test with the standard error (SE) of the observed outcome.

## 3. Results

### 3.1. Literature search

**Figure 1:**
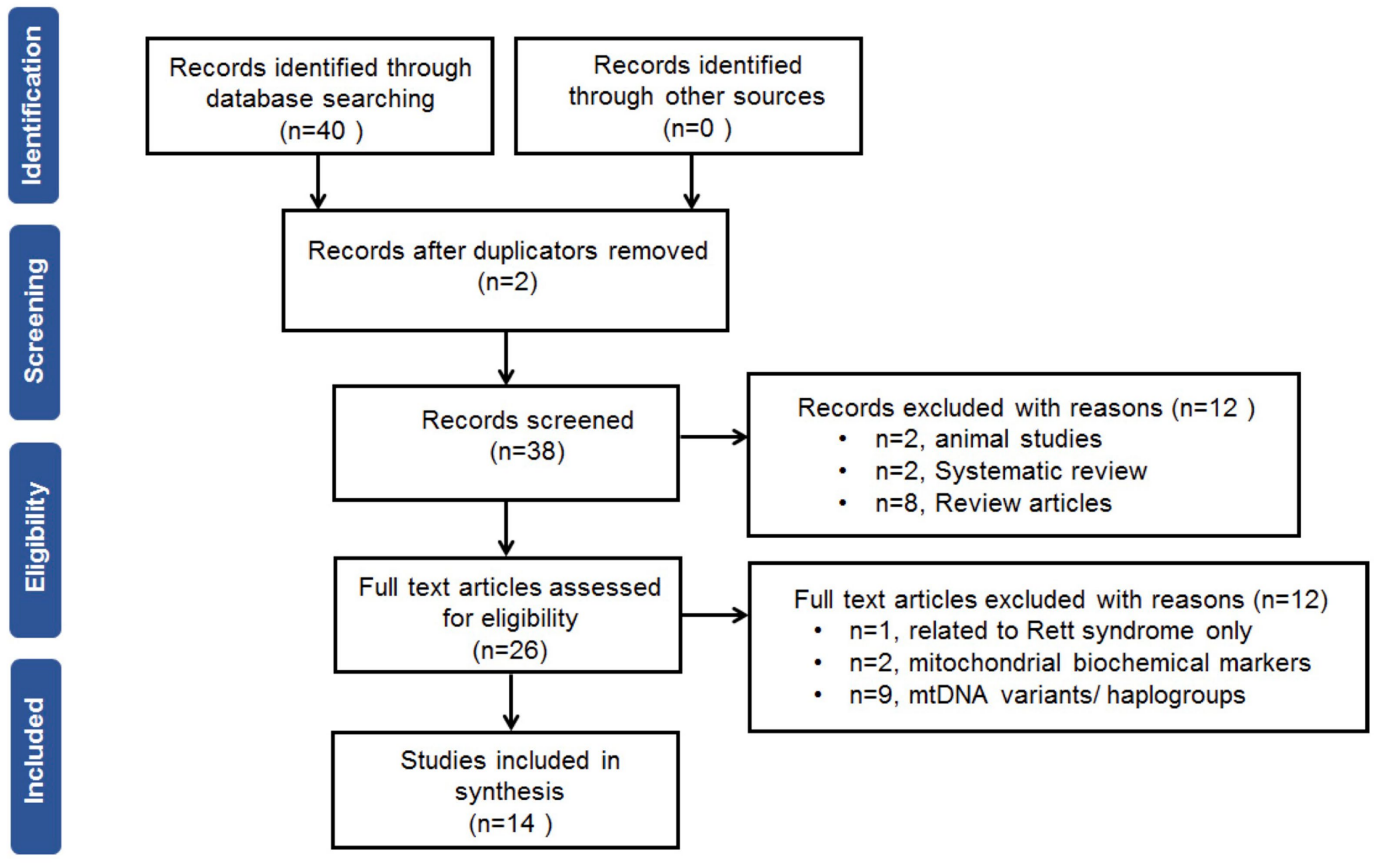
Preferred reporting items for systematic review and meta-analysis flow chart.

The search of the electronic databases retrieved 38 studies after removal of duplicates. After screening of the titles and abstracts, fourteen full-text studies were eligible for the meta-analysis. The flow chart of the search and selection processes with a PRISMA (Preferred Reporting Items for Systematic Reviews and Meta-Analyses) is shown in Figure 1.

### 3.2. Data extraction

Two authors extracted the data independently with a standard extraction table. The extracted information include first author’s name, publication year, disease, subject’s ethnicity, sample used, sample size, age, gender, age of disease onset, disease duration, clinical assessment, and outcome.

### 3.3. Characteristics of the included studies

A total of 14 studies involving 666 cases and 585 controls were used in the current systematic review and meta-analysis. Table 1 illustrates the characteristics of included studies. Eleven studies each focused on ASD (26–29, 37–42, 44), while three studies each focused on ADHD (30, 31, 43). There were seven studies recruited Caucasian subjects (27–29, 35, 37–39, 42), five studies recruited Asian subjects (26, 30, 31, 40, 43), and two study recruited subjects from mixed ethnic backgrounds (41, 44). The age and gender of participants varied between studies. The percentages of males were higher than females in most of the studies, while the participants were all males in one study (43), or all females in another study (28). Age of participants was within a range between 2 to 13 years old in 12 studies (26, 28, 30, 31, 37–44). Age of disease onset and disease duration were only mentioned in one study (40). For the diagnosis of ASD, the studies have used clinical examinations using the Autism Diagnostic Inventory-Revised (ADI-R) (45), and the Autism Diagnostic Observation Schedule (ADOS) (46) according to the Diagnostic and Statistical Manual of Mental Disorders, fifth edition (DSM-5) diagnostic criteria-based structured interview for autism (1). The severity of autism was assessed by the Childhood Autism Rating Scale (CARS), a 15-items behavioral rating scale (47) as well as the Autistic Behavior Checklist (ABC), a 57-items atypical behaviors rating scale (48). ASD was also assessed by the clinician-rated Ohio Autism Clinical Impression Scale-Severity (OACIS-S) (49) and the Social Responsiveness Scale-2 (SRS) (50). For the diagnosis of ADHD, the studies have used clinical assessment using the semi-structured interview-Kiddie Schedule for Affective Disorders and Schizophrenia Present and Lifetime Version (K-SADS-PL) according to Diagnostic and Statistical Manual of Mental Disorders (DSM-5) criteria (51).

**Table 1:** Characteristics of the included studies.

No.	Study Ref.	Author/Year	Disease	Ethnicity	Sample size Cases/controls	Age (years) Cases/controls	Gender		Age of Disease onset (years)	Disease duration (years)	Clinical assessment
							Female (%) Cases/controls	Male (%) Cases/controls			
1	Giulivi et al. (37)	Giulivi/2010	ASD	Caucasian	10/10	2–5	10/10	90/90	NP	NP	ADI-R, ADOS
2	Gu et al. (38)	Gu/2013	ASD	Caucasian	14/12	10.36 ± 1.46/10.83 ± 1.72	3/3	11/9	NP	NP	DSM-IV
3	Tang et al. (29)	Tang/2013	ASD	Caucasian	20/25	3–60/2–65	8/12	90/88	NP	NP	DSM-IV, ADI-R
4	Napoli et al. (39)	Napoli/2014	ASD	Caucasian	10/10	2–5	10/10	90/90	NP	NP	ADI-R, ADOS
5	Chen et al. (40)	Chen/2015	ASD	Asian	78/83	3.78/3.9	11.5/19.2	88.4/80.7	2.05 ± 0.7	2.49 ± 1.2	CARS, ABC
6	Yoo et al. (26)	Yoo/2016	ASD	Asian	100/100	8.2/7.6	17/39	83/61	NP	NP	DSM-IV, ADI-R
7	Wong et al. (41)	Wong/2016	ASD	Mixed	66/46	3.9 ± 0.1/3.5/±0.1	15.4/12.9	84.6/87.1	NP	NP	ADOS
8	Valiente-Palleja et al. (27)	ValientePalleja`/2018	ASD	Caucasian	122/112	40.7 ± 8.3/42.4 ± 11.4	34.4/55.4	65.5/44.6	NP	NP	DSM-IV, ADI-R
9	Carrasco et al. (42)	Carrasco/2019	ASD	Caucasian	12/12	6–13	8.3/25	91.6/75	NP	NP	DSM-IV
10	Kim et al. (43)	Kim/2019	ADHD	Asian	70/70	9.8 ± 2.6/10 ± 2.6	–	44/44	NP	NP	K-SADS-PL DSM-IV
11	Singh et al. (28)	Singh/2019	ASD	Caucasian	12/9	5.14 ± 1.5/6.60 ± 2.1	25/44	–	NP	NP	OACIS-S, ABC, SRS
12	Öğütlü et al. (30)	Öğütlü/2020	ADHD	Asian	56/56	9.88 ± 2.60/9.96 ± 2.78	37.5/37.5	62.5/62.5	NP	NP	K-SADS-PL DSM-IV
13	Bam et al. (44)	Bam/2021	ASD	Mixed	68/40	6–12	NP	NP	NP	NP	ADOS
14	Öğütlü et al. (31)	Öğütlü/2021	ADHD	Asian	28	10.0 ± 3.4/9.7 ± 2.1	64.3/28.6	35.7/71.4	NP	NP	K-SADS-PL DSM-IV

ASD, autism spectrum disorder; ADHD, attention deficit hyperactivity disorder; NP, not provided; CARS, Childhood Autism Rating Scale; ABC, Autistic Behavior Checklist; DSM-IV, Diagnostic and Statistical Manual of Mental Disorders – fourth edition; ADI-R, Autism Diagnostic Interview-Revised; ADOS, Autism Diagnostic Observation Schedule; OACIS-S, clinician-rated Ohio Autism Clinical Impression Scale–Severity; SRS, Social Responsiveness Scale; K-SADS-PL, Schedule for Affective Disorders and Schizophrenia for School Age Children Present and Lifetime version; ADOS, Autism Diagnostic Observation Schedule; SRS, Social Responsiveness Scale.

### 3.4. Description and outcome of the included studied

Description and outcome of the included studies are summarized in Table 2. Of the 14 studies, four focused solely on mtDNA-cn (26, 30, 31, 40). The reaming studies evaluated the mtDNA-cn as well as other effects such as mtDNA deletion (37, 39, 41, 44), mtDNA variations/polymorphisms (27, 42), mitochondrial activity (26, 29, 37–39), mitochondrial gene expression or oxidative stress (37, 39, 42), and mtDNA methylation (43, 44). For mtDNA-cn measurement, ten studies collected blood samples (26–28, 30, 31, 37, 39–41, 43), two studies used brain tissue samples (29, 38), and two studies used oral samples (42, 44). All of the studies used quantitative real-time PCR (qPCR) to determine the amount of mtDNA relative to nuclear DNA (nDNA) by obtaining the mtDNA/nDNA ratio and expressed the average mtDNA-cn per cell. Total DNA was extracted using available commercial DNA extraction kits. Different sets of primers of mitochondrial genes and nuclear genes were used for determination of mtDNA-cn in qPCR. Compared to controls, ten studies reported high mtDNA-cn in cases with neurodevelopmental disorders including eight studies on ASD (26, 37–42, 44), and three studies on ADHA (30, 31, 43). Whereas two studies reported low mtDNA-cn in ASD cases compared to controls (27, 28), and one study found no significant difference in mtDNA-cn between cases with ASD and controls (29). Regarding the outcome, all studies revealed that altered mtDNA-cn was associated with mitochondrial dysfunction in ASD and ADHD. In ASD, the study by Yoo et al. (26) revealed a significant correlation between elevated mtDNA-cn and clinical phenotypes for language and communication. Whereas the study by Chen et al. (40) showed no significant association between higher mtDNA-cn and clinical features including CARS and ABC scores, suggesting that dysfunctional mitochondria may be related to autism subtypes. A clear link between higher mtDNA-cn and oxidative stress was reported in ASD in three studies (37, 39, 42). In ADHD, all of the studies revealed an important role of elevated mtDNA-cn in the eitiology and/or pathophysiology of the disease (30, 31, 43).

**Table 2:** Description and outcome of the included studies.

No.	Study Ref.	Author/Year	Disease	Aim	Sample used	Method of mtDNA-cn assessment	mtDNA gene	nDNA gene	Finding	Conclusion
1	Giulivi et al.(37)	Giulivi/2010	ASD	mtDNA-cn and deletions, and mitochondrial activity	White blood cells	qPCR	ND1, ND4	PK, APP	Higher mtDNA-cn and mtDNA deletions in ASD patients compared to controls; low NADH, low activity of complex I, high plasma pyruvate levels and lower pyruvate dehydrogenase activity; higher oxidative stress in ASD patients compared to controls	Mitochondrial dysfunction, mtDNA overreplication, and mtDNA deletions are more likely to occur in ASD have than typically developing children
2	Gu et al. (38)	Gu/2013	ASD	mtDNA-cn and mitochondrial activity	Frontal cortex tissue	qPCR	ND1, ND4, CYTB	PK	Higher mtDNA-cn in ASD patients than in controls; defects in complexes I, III and V, and reduced PDH activity in ASD patients compared to controls	Mitochondrial dysfunction in the brain is associated with ASD
3	Tang et al. (29)	Tang/2013	ASD	mtDNA-cn and mitochondrial activity	Temporal cortex tissue	qPCR	12S rRNA	RNAseP	No differences in either mtDNA-cn or levels of the mitochondrial gene transcription factor TFAM or cofactor PGC1α in ASD patients and controls; altered mitochondrial dynamics, protein levels of mitochondria respiratory chain protein complexes, decreased Complex I and IV activities; decreased mitochondrial antioxidant enzyme SOD2; and increased oxidative DNA damage and mitochondrial membrane mass in ASD patients	Mitochondrial dysfunction in early childhood ASD
4	Napoli et al. (39)	Napoli/2014	ASD	mtDNA-cn and deletions, and mitochondrial activity	White blood cells	qPCR	ND1, ND4	PK, APP	Higher oxidative stress in patients with autism; higher rates of mitochondrial ROS production; higher mtDNA-cn; and increased mtDNA deletions	Molecular network linking mitochondrial function, OXPHOS and the inflammation/immune response in ASD
5	Chen et al. (40)	Chen/2015	ASD	mtDNA-cn	Peripheral blood	qPCR	mtDNA primers (L39, H475)	HBB	Higher mtDNA-cn in ASD patients compared to controls; no significant correlations between mtDNA-cn and clinical features including paternal age, maternal age, age of onset, illness of duration, CARS score and ABC score in childhood autism	Elevated mtDNA-cn is associated with ASD, indicating mitochondrial dysfunction in children with autism
6	Yoo et al. (26)	Yoo/2016	ASD	mtDNA-cn	Peripheral blood	qPCR	ND1, ND4, CYTB	PK	Higher mtDNA-cn in ASD patients than in unaffected sibs; significant correlations between mtDNA-cn and clinical phenotypes for language and communication in ASD	Mitochondrial dysfunction and elevated mtDNA-cn in ASD are related to the phenotype for communication
7	Wong et al. (41)	Wong/2016	ASD	mtDNA-cn and deletions, and p53 gene copy ratios	Peripheral blood mononuclear cell	qPCR	ND1, CYTB	PK	Higher mtDNA-cn in ASD patients than in controls; higher incidence of mtDNA deletions in ASD patients and their fathers	Genome instability and altered mtDNA-cn in ASD
8	Valiente-Palleja et al. (27)	Valiente-Palleja`/2018	ASD	mtDNA-cn and mtDNA mutations	White blood cells		ND1, ND4	NP	Lower mtDNA-cn in ASD and ID patients than in controls; a total of 28.6% of ASD and 30.5% of ID subjects carried at least one putative pathogenic mtDNA mutation	Mitochondrial dysfunction in ASD and ID
9	Carrasco et al. (42)	Carrasco/2019	ASD	mtDNA-cn, oxidative stress, complexes, polymorphisms and gene expression of mitochondrial SOD2	Buccal cells	qPCR	tRNA-Leu	B2M	Higher mtDNA-cn in ASD compared to controls, enhanced ROS generation; significantly lower levels of respiratory complex I and decreased complex I and IV activities; presence of C47T polymorphism in SOD2 gene results in Ala16Val change could affect the transport of the SOD2 enzyme to the mitochondrial matrix and increases oxidative stress	Involvement of mitochondrial biology in the development of ASD
10	Kim et al. (43)	Kim/2019	ADHD	mtDNA-cn, methylation ratio of the D-loop region and PPARGC1A	Peripheral blood	qPCR	CYTB	PK	Higher mtDNA-cn in ADHD patients than in controls; decreased methylation ratio of PPARGC1A in ADHD	Mitochondrial dysfunction plays a role in the pathophysiology of ADHD
11	Singh et al. (28)	Singh/2019	ASD	mtDNA-cn and mitochondrial activity	Peripheral blood mononuclear cell	qPCR	ND4, CYTB	PK	Lower mtDNA-cn in ASD than in controls; higher maximal oxygen consumption rate, maximal respiratory capacity and reserve capacity in ASD and DR children than in ASD without DR; association of Coupling Efficiency and Maximal Respiratory Capacity with disruptive behaviors	Mitochondrial function is related to ASD symptoms and a potential mitochondrial therapeutic target in ASD
12	Öğütlü et al. (30)	Öğütlü/2020	ADHD	mtDNA-cn	Peripheral blood	qPCR	ND1	HBB	Higher mtDNA-cn in ADHD patients than in controls	Mitochondrial dysfunction is related to the etiopathogenesis of ADHD
13	Bam et al. (44)	Bam/2021	ASD	mtDNA-cn, DNA methylation of PGC-1α and mtDNA deletion	Buccal cells	qPCR	ND1	B2M	Higher mtDNA-cn in ASD patients than in control; methylation at the PGC-1α promoter can lead to mtDNA deletion and associated with high mtDNA-cn	Mitochondrial dysfunction in ASD
14	Öğütlü et al. (31)	Öğütlü/2021	ADHD	mtDNA-cn	Peripheral blood	qPCR	ND1	HBB	High mtDNA-cn in ADHD patients regardless of treatment	Mitochondrial dysfunction plays a role in pathophysiology of ADHD

ASD, autism spectrum disorder; ADHD, attention deficit hyperactivity disorder; mtDNA-cn, mitochondrial DNA copy number; qPCR, quantitative PCR; nDNA, nuclear DNA; ND1, NADH dehydrogenase 1; ND4, NADH dehydrogenase 4, CYTB, cytochrome b; HBB, beta-globin; PK; pyruvate kinase; APP, amyloid β A4 precursor protein; B2M, beta-2-microglobulin; NP, not provided.

### 3.5. Relationship between mtDNA-cn and ASD/ADHD in overall and stratified meta-analyses

**Figure 2:**
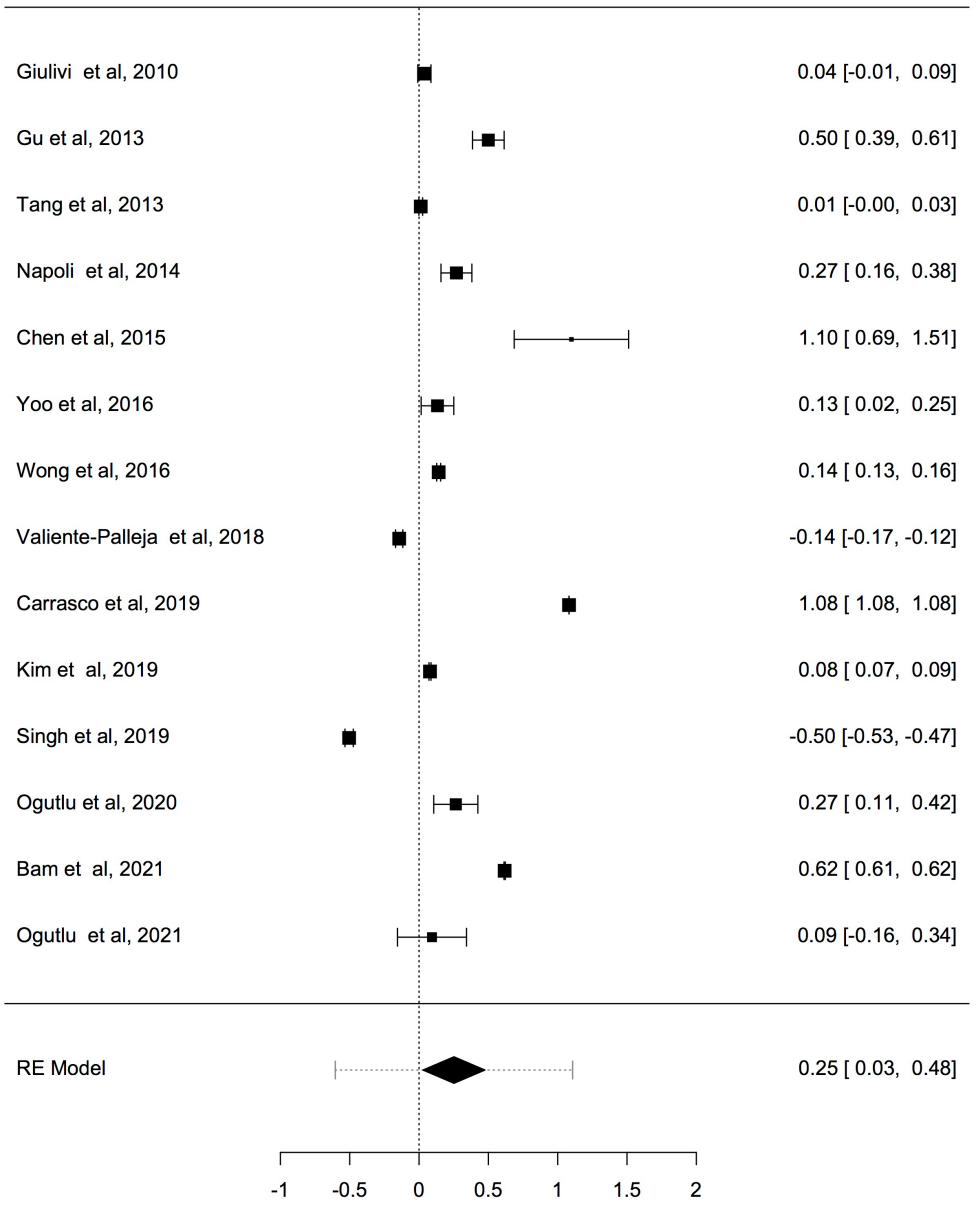
Forest plot overall meta-analysis of mtDNA-cn in ASD/ADHD patients and controls (*p* = 0.0275).

Fourteen studies reported differences in mtDNA-cn in ASD and ADHD patients and controls were included in the meta-analysis. The mtDNA-cn was quantified using blood samples in 10 studies and using brain/oral samples in four studies. First we analysed all 14 studies reported changes in mtDNA-cn in ASD/ADHD patients (*n* = 666) and controls (*n* = 585) regardless of the used biological samples (*k* = 14). The overall meta-analysis of pooled studies by dichotomizing mtDNA-cn into high and low, showed a significant relationship between increased mtDNA-cn and ASD/ADHD (Figure 2). Geometric mean difference = 0.252 (95% CI: 0.03 to 0.48); Z = 2.205; *p* = 0.027. Heterogeneity, I^2^ = 99.9%, *p* < 0.0001. I^2^ values showed very large heterogeneity across studies (Table 3). According to the studentized residuals examination, none of the studies had a value > ± 2.9137, indicating no outliers in the context of this model. According to the Cook’s distances, none of the studies was overly influential.

**Table 3:** Random-effects model and heterogeneity statistics of overall meta-analysis of mtDNA-cn in ASD/ADHD patients and controls (*k* = 14).

Random-effects model							
	Estimate	SE	Z	95% CI Lower Bound	95% CI Upper Bound	*p*-value	
Intercept	0.25	0.11	0.20	0.03	0.48	0.0275	
Heterogeneity statistics							
Tau	Tau^2^	I^2^	H^2^	R^2^	df	Q	*p*-value
0.42	0.1777 (SE = 0.0716)	99.9%	8234.3		13.00	171751.8	< 0.001

**Figure 3:**
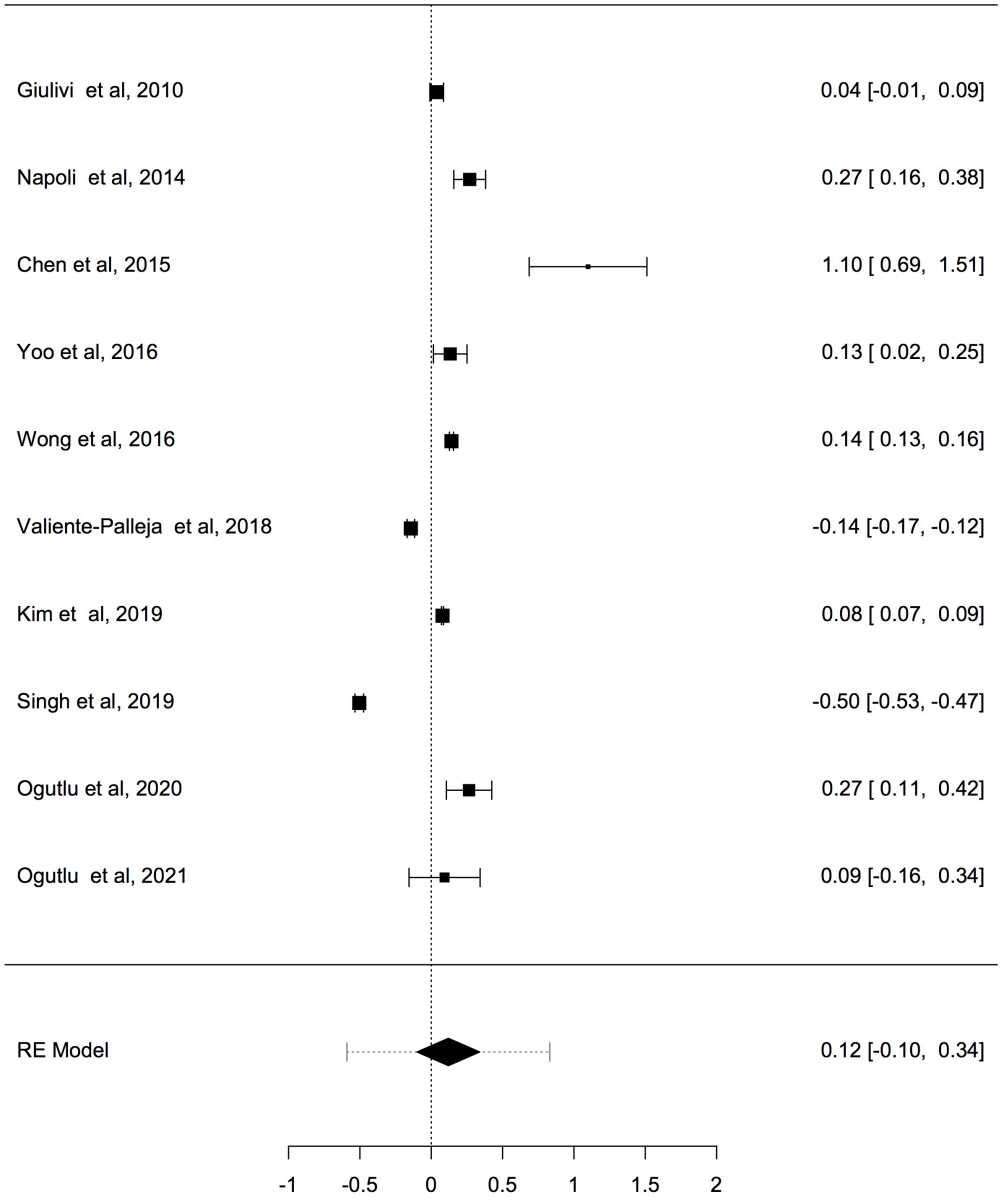
Forest plot meta-analysis of mtDNA-cn in ASD/ADHD patients and controls based on blood samples (peripheral blood, leukocytes, and PBMCs), (*p* = 0.284).

**Figure 4:**
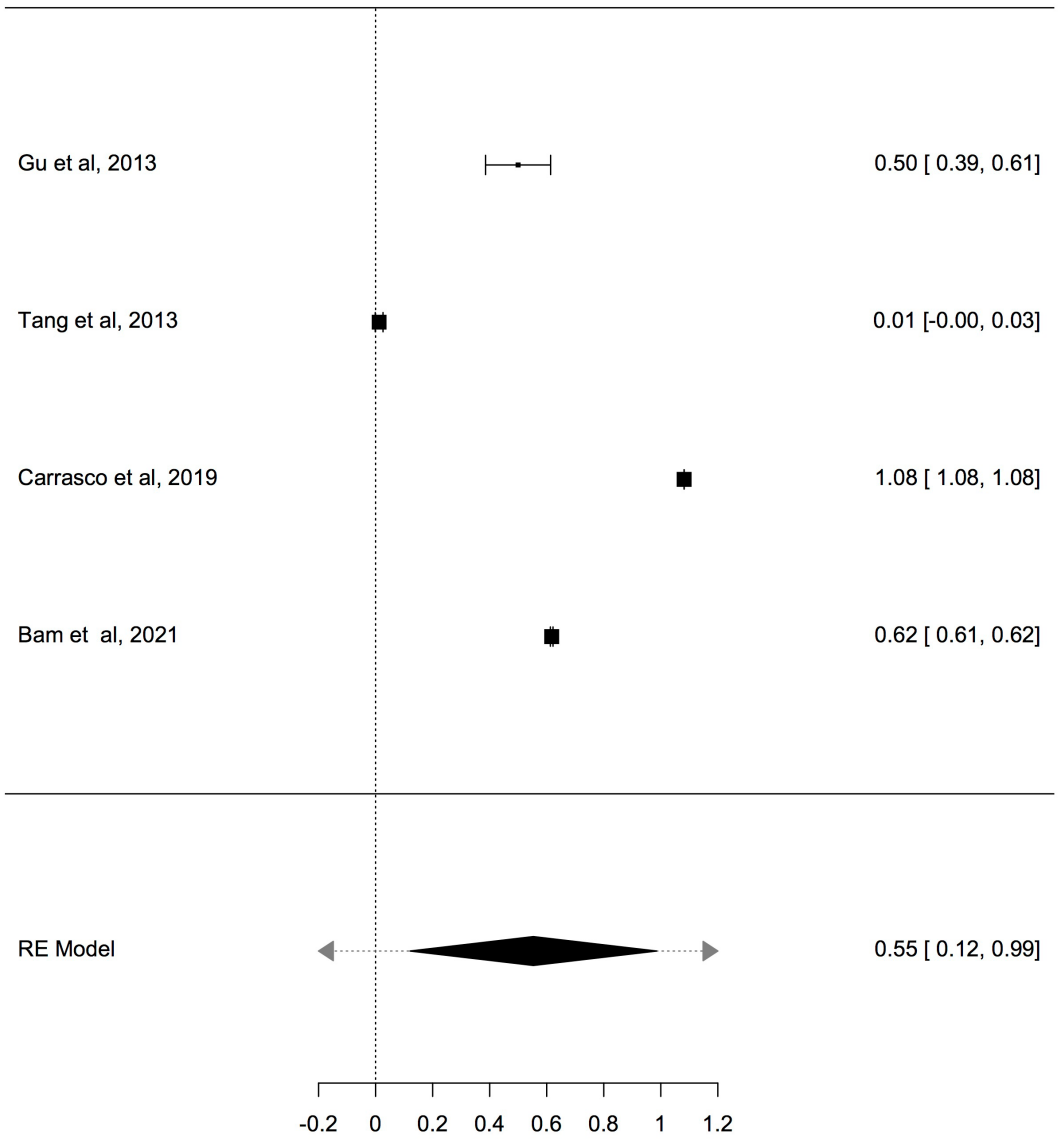
Forest plot meta-analysis of mtDNA-cn in ASD/ADHD patients and controls based on non-blood samples (brain and oral tissues), (*p* = 0.012).

Previous studies have shown that the mtDNA-cn varies widely across cell types and depends on the energy demand and physiological condition (16, 17). Therefore, we analysed studies reported changes in mtDNA-cn in ASD/ADHD patients and controls according to biological samples. To reflect the biological differences between mtDNA-cn in the systemic circulation vs. other tissues (brain and oral tissues), mtDNA-cn measured in peripheral blood, leukocytes, and mononuclear cells (PBMCs) were combined for comparison under the category “blood based mtDNA” and mtDNA-cn measured in the brain and oral tissues were combined under the category “non-blood based mtDNA.” In this comparison, ten studies including 552 patients and 496 controls were blood based and four studies including 114 patients and 89 controls were non-blood based. The mtDNA-cn in blood based samples of patients and controls did not reach statistical significance (Figure 3). Geometric mean difference = 0.1200 (95% CI: −0.0995 to 0.339); Z = 1.0713, *p* = 0.284, and a larger heterogeneity was observed (I^2^ = 99.8%, *p* < 0.001) (Table 4). Conversely, the mtDNA-cn in non-blood based samples was significantly higher in patients than in controls (Figure 4). Geometric mean difference = 0.5531 (95% CI: 0.1208 to 0.9854); Z = 2.5075, *p* = 0.0122, with a large heterogeneity (I^2^ = 99.9%, *p* < 0.001) (Table 5).

**Table 4:** Random-effects model and heterogeneity statistics of meta-analysis of mtDNA-cn in ASD/ADHD patients and controls based on blood samples (peripheral blood, leukocytes, and PBMCs) (*k* = 10).

Random-effects model							
	Estimate	SE	Z	95% CI Lower Bound	95% CI Upper Bound	*p*-value	
Intercept	0.21	0.11	1.07	−0.10	0.34	0.284	
Heterogeneity statistics							
Tau	Tau^2^	I^2^	H^2^	R^2^	df	Q	*p*-value
0.34	0.1119 (SE = 0.059)	99.8%	648.5		9.00	1789.9	< 0.001

**Table 5:** Random-effects model and heterogeneity statistics of meta-analysis of mtDNA-cn in ASD/ADHD patients and controls based on non-blood samples (*k* = 4).

Random-effects model							
	Estimate	SE	Z	95% CI Lower Bound	95% CI Upper Bound	*p*-value	
Intercept	0.55	0.22	2.51	0.12	0.99	0.012	
Heterogeneity statistics							
Tau	Tau^2^	I^2^	H^2^	R^2^	df	Q	*p*-value
0.44	0.1937 (SE = 0.159)	99.9%	25689.57		3.00	62146.4	< 0.001

**Figure 5:**
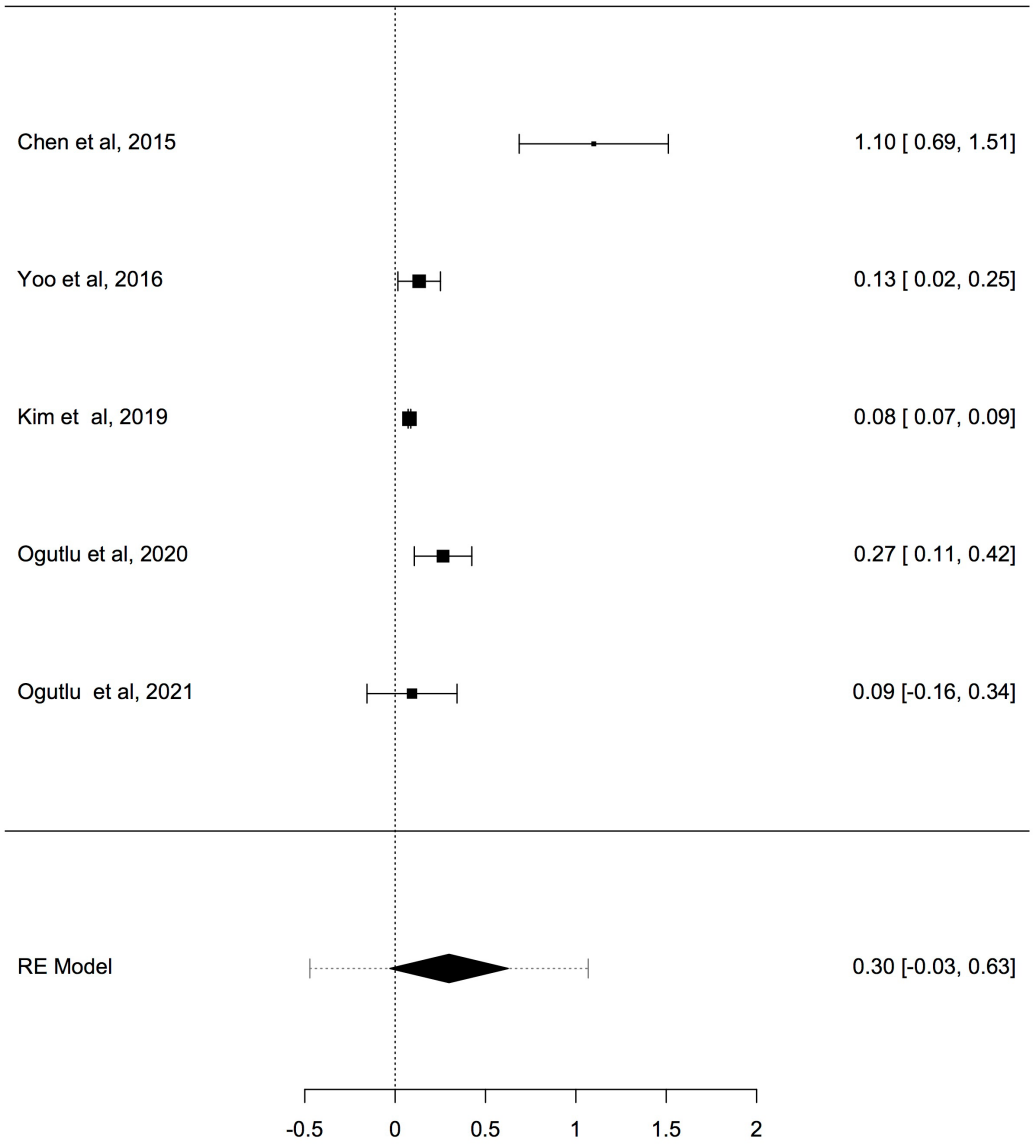
Forest plot meta-analysis of mtDNA-cn in ASD/ADHD patients and controls based on peripheral blood samples only (*p* = 0.074).

To adjust for blood cell compositions as potential confounds, further analysis was carried out only on studies reported changes in mtDNA-cn in peripheral blood samples of ASD/ADHD patients and controls (*k* = 5). The results showed no significant difference in peripheral blood mtDNA-cn between patients and controls (Figure 5). Geometric mean difference = 0.2983 (95% CI: −0.0286 to 0.6252); Z = 1.7883, *p* = 0.074, and a substantial level of heterogenicity was observed (I^2^ = 96.9%, *p* < 0.001) (Table 6).

**Table 6:** Random-effects model and heterogeneity statistics of meta-analysis of mtDNA-cn in ASD/ADHD patients and controls based on peripheral blood samples only (*k* = 5).

Random-effects model							
	Estimate	SE	Z	95% CI Lower Bound	95% CI Upper Bound	*p*-value	
Intercept	0.30	0.17	1.79	−0.03	0.63	0.074	
Heterogeneity statistics							
Tau	Tau^2^	I^2^	H^2^	R^2^	df	Q	*p*-value
0.36	0.1266 (SE = 0.098)	97%	33.32		4.00	29.5	< 0.001

**Figure 6:**
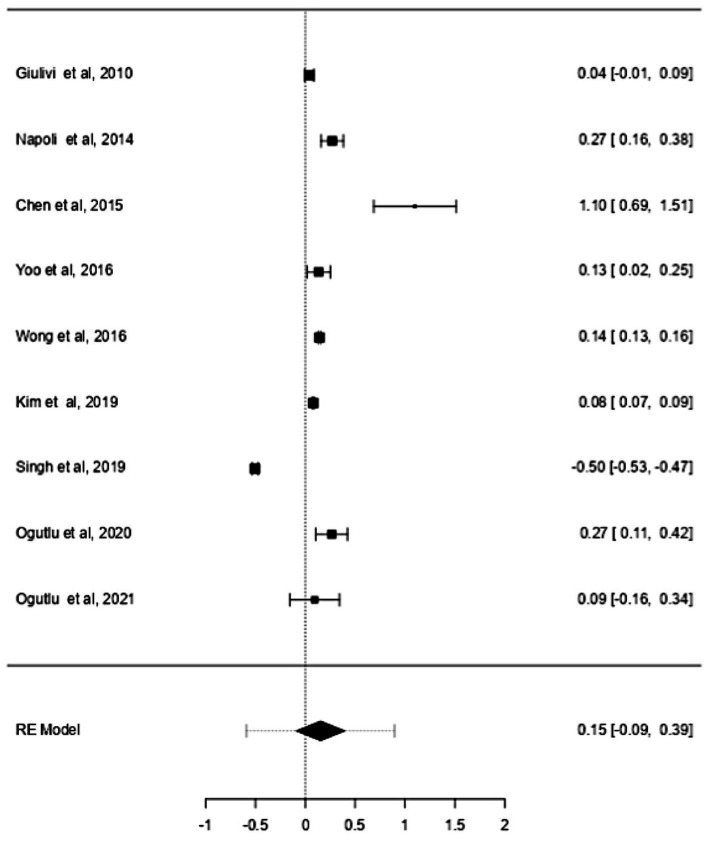
Forest plot meta-analysis of mtDNA-cn in age-matched ASD/ADHD patients and controls based on blood samples (*p* = 0.214).

**Figure 7:**
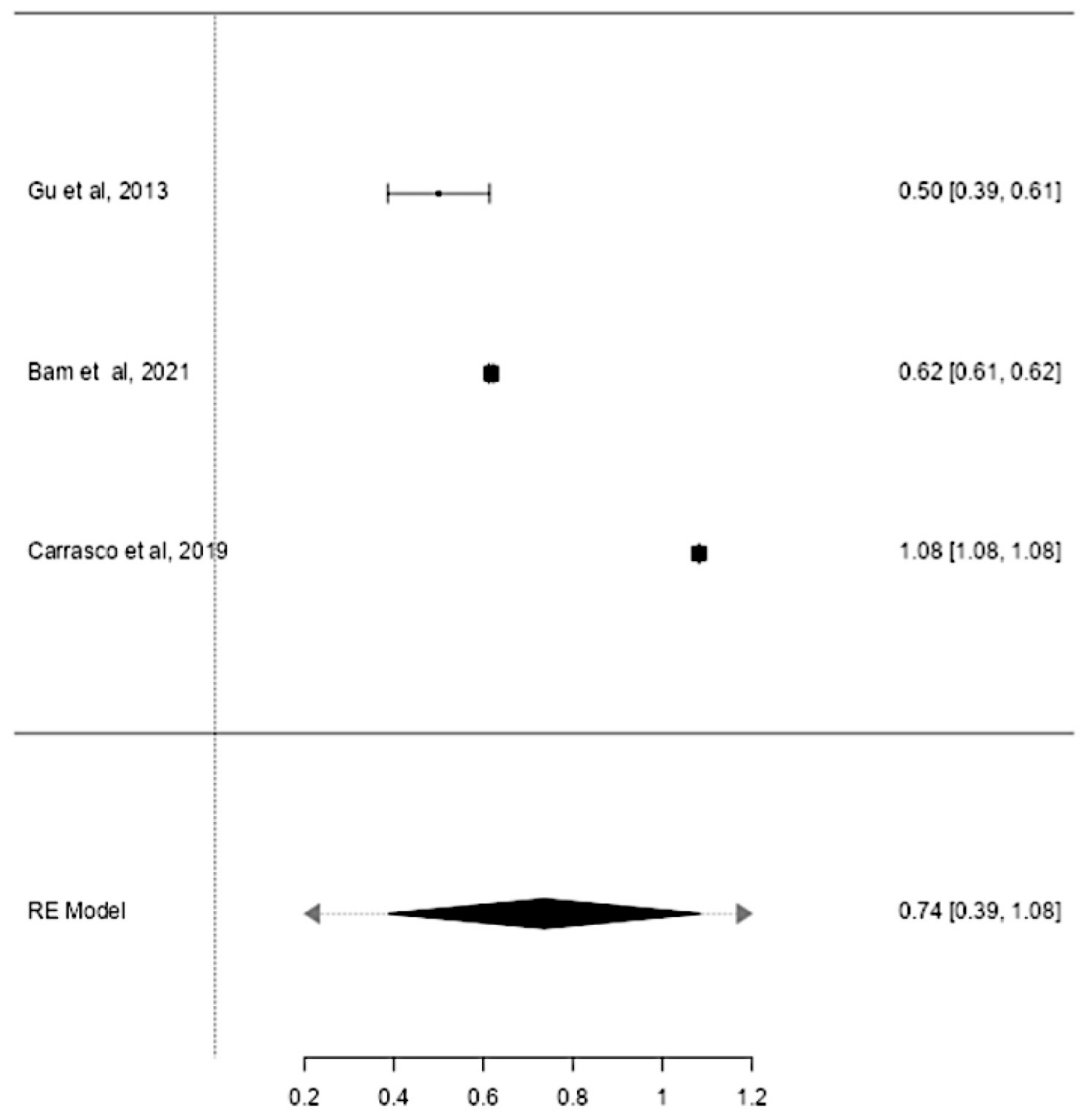
Forest plot meta-analysis of mtDNA-cn in age-matched ASD/ADHD patients and controls based on non-blood samples (*p* < 0.001).

Studies have also shown that the mtDNA-cn may differ according to the age of individuals (18, 19). Thus, further stratified analysis was conducted on studies reported differences in the mtDNA-cn in patients with ASD/ADHD and controls within a range age between 2–13 years old. In studies conducted using blood samples (*k* = 9), the mtDNA-cn did not reach statistical significance (Figure 6). Geometric mean difference = 0.15 (95% CI: −0.099 to 0.33); Z = 1.24, *p* = 0.214, and a larger heterogeneity was observed (I^2^ = 99.85%, *p* < 0.001) (Table 7). On the other hand, the mtDNA-cn in non-blood based samples (*k* = 3) was significantly higher in patients than in controls (Figure 7). Geometric mean difference = 0.74 (95% CI: 0.39 to 1.08); Z = 4.13, *p* < 0.001, with a large heterogeneity (I^2^ = 99.9%, *p* < 0.001) (Table 8).

**Table 7:** Random-effects model and heterogeneity statistics of meta-analysis of mtDNA-cn in ASD/ADHD patients and controls based on of age-matched, blood samples (*k* = 9).

Random-effects model							
	Estimate	SE	Z	95% CI Lower Bound	95% CI Upper Bound	*p*-value	
Intercept	0.15	0.12	1.24	−0.09	0.69	0.214	
Heterogeneity statistics							
Tau	Tau^2^	I^2^	H^2^	R^2^	df	Q	*p*-value
0.36	0.1282 (SE = 0.07)	99.8%	652.07		8.00	1558.5	<0.001

**Table 8:** Random-effects model and heterogeneity statistics of meta-analysis of mtDNA-cn in ASD/ADHD patients and controls based of age-matched, non-blood samples (*k* = 3).

Random-effects model							
	Estimate	SE	Z	95% CI Lower Bound	95% CI Upper Bound	*p*-value	
Intercept	0.174	0.18	4.13	0.39	1.08	< 0.001	
Heterogeneity statistics							
Tau	Tau^2^	I^2^	H^2^	R^2^	df	Q	*p*-value
0.31	0.094 (SE = 0.095)	99.9%	16,741		2.00	38,458	<0.001

**Figure 8:**
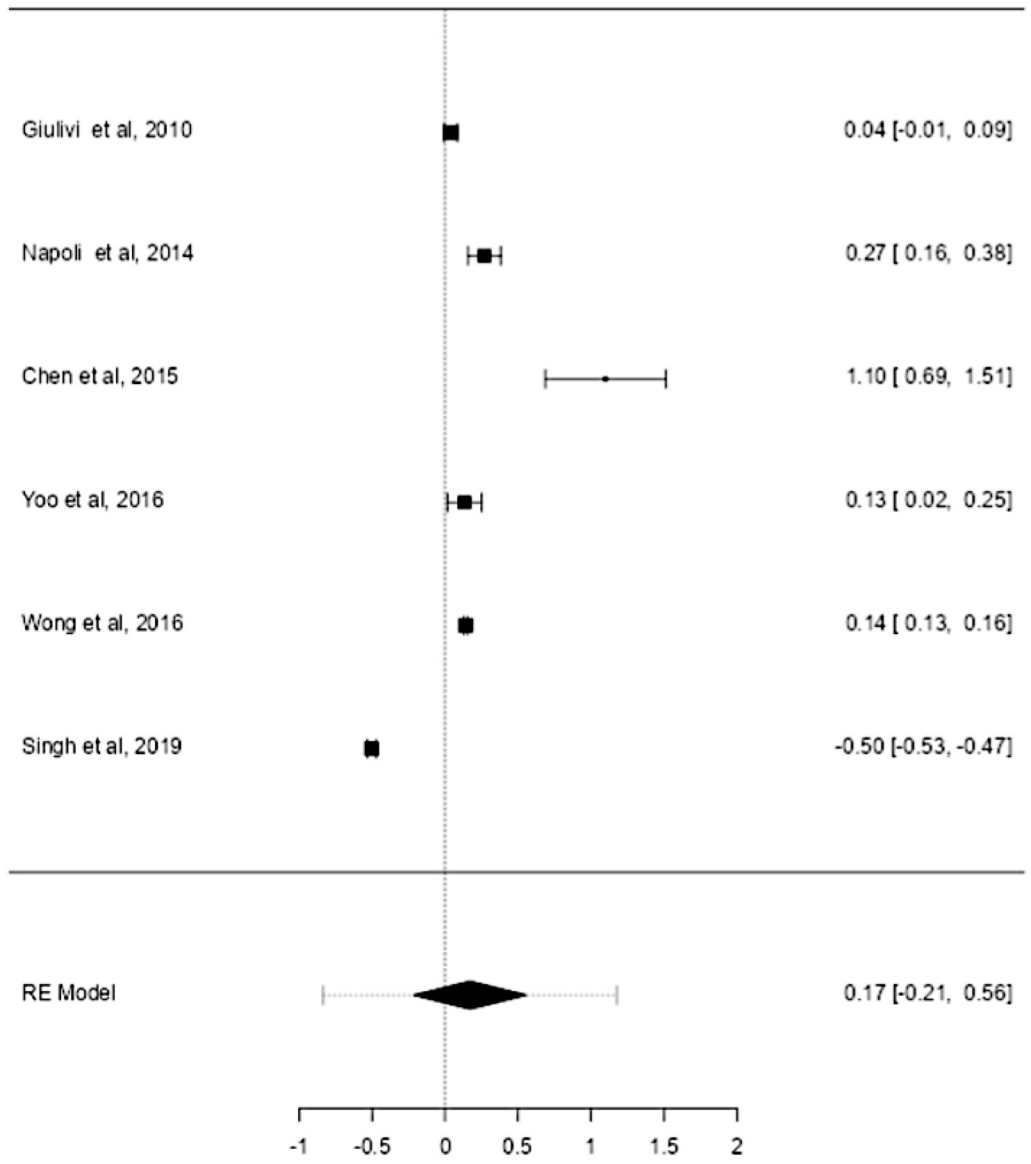
Forest plot meta-analysis of mtDNA-cn in age-matched ASD patients and controls based on blood samples (peripheral blood, leukocytes, and PBMCs) (*p* = 0.385).

**Figure 9:**
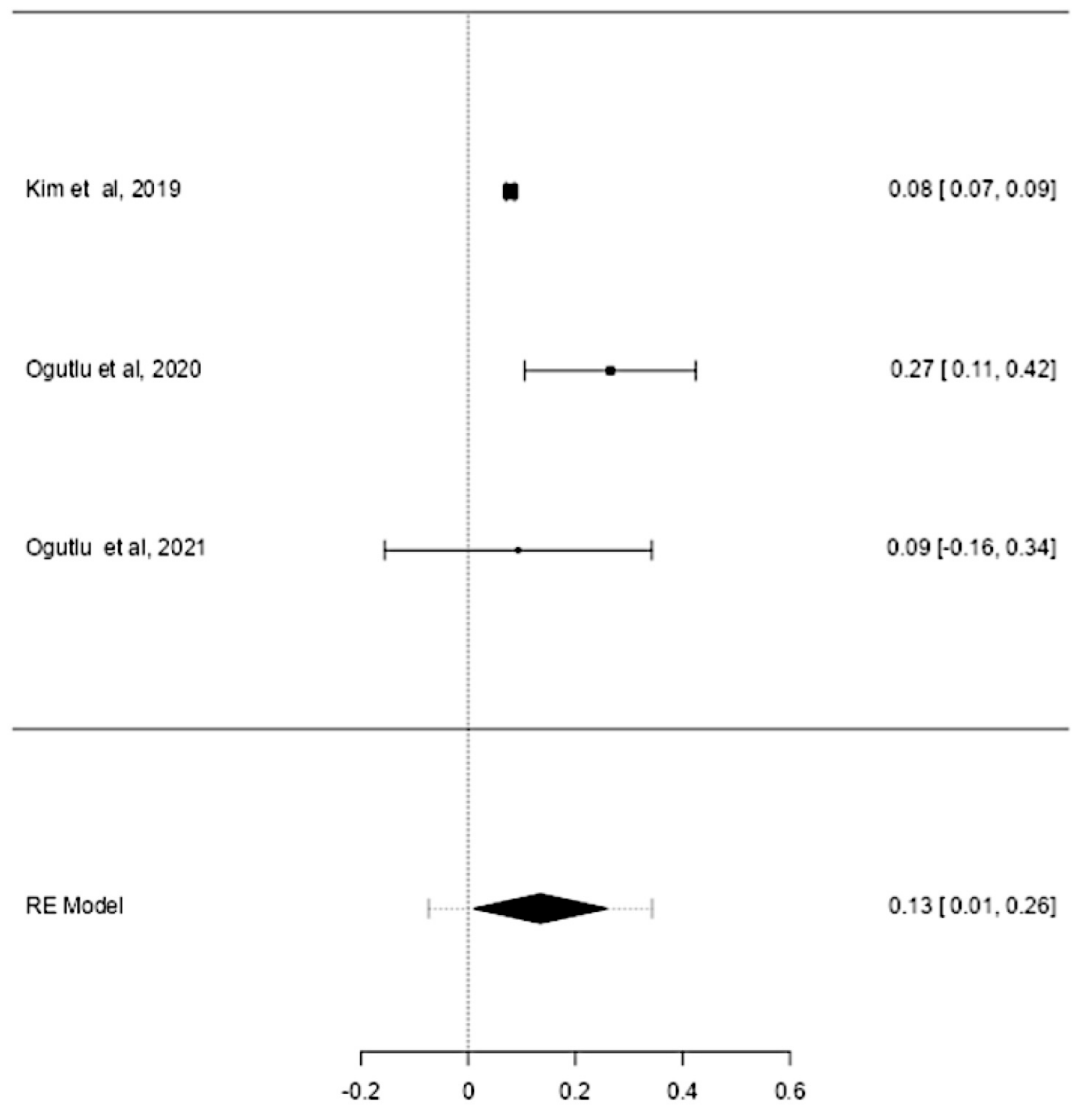
Forest plot meta-analysis of mtDNA-cn in age-matched ASD patients and controls based on blood samples (peripheral blood) (*p* = 0.033).

ASD and ADHD share some clinical features (5, 6), which may be due to shared genetic factors and other influences (7–9). We sought to determine changes in mtDNA-cn in each disorder. Accordingly, sub-analysis was carried out by stratifying studies on ASD only (*k* = 6) or ADHD only (*k* = 3). In this sub-analysis, only blood samples were considered since all ADHD studies used blood to measure mtDNA-cn. Notably, measurement of mtDNA-cn in ASD patients was done using peripheral blood, leukocytes, and PBMCs, whereas only peripheral blood was used to measure the mtDNA-cn in ADHD patients. The results showed no significant difference in blood mtDNA-cn between ASD patients and controls (Figure 8). Geometric mean difference = 0.17 (95% CI: −0.021 to 0.56); Z = 0.87, *p* = 0.385, and a substantial level of heterogenicity was observed (I^2^ = 99.7%, *p* < 0.001) (Table 9). On the other hand, a significant increase in blood mtDNA-cn was found between ADHD patients and controls (Figure 9). Geometric mean difference = 0.13 (95% CI: 0.01 to 0.26); Z = 2.14, *p* = 0.033, with a low level of heterogenicity (I^2^ = 60.8%, *p* = 0.072) (Table 10).

**Table 9:** Random-effects model and heterogeneity statistics of meta-analysis of mtDNA-cn in ASD patients based on peripheral blood, leukocytes, and PBMCs samples (*k* = 6).

Random-effects model							
	Estimate	SE	Z	95% CI Lower Bound	95% CI Upper Bound	*p*-value	
Intercept	0.174	0.20	0.87	−0.21	0.56	0.385	
Heterogeneity statistics							
Tau	Tau^2^	I^2^	H^2^	R^2^	df	Q	*p*-value
0.47	0.2246 (SE = 0.145)	99.8%	478.2		5.00	1,504	<0.001

**Table 10:** Random-effects model and heterogeneity statistics of meta-analysis of mtDNA-cn in ADHD patients based on peripheral blood samples (*k* = 3).

Random-effects model							
	Estimate	SE	Z	95% CI Lower Bound	95% CI Upper Bound	*p*-value	
Intercept	0.13	0.06	2.14	0.01	0.26	0.033	
Heterogeneity statistics							
Tau	Tau^2^	I^2^	H^2^	R^2^	df	Q	*p*-value
0.09	0.0073 (SE = 0.013)	60.87%	2.56		2.00	5.25	0.072

### 3.6. Assessment of the risk of bias

**Figure 10:**
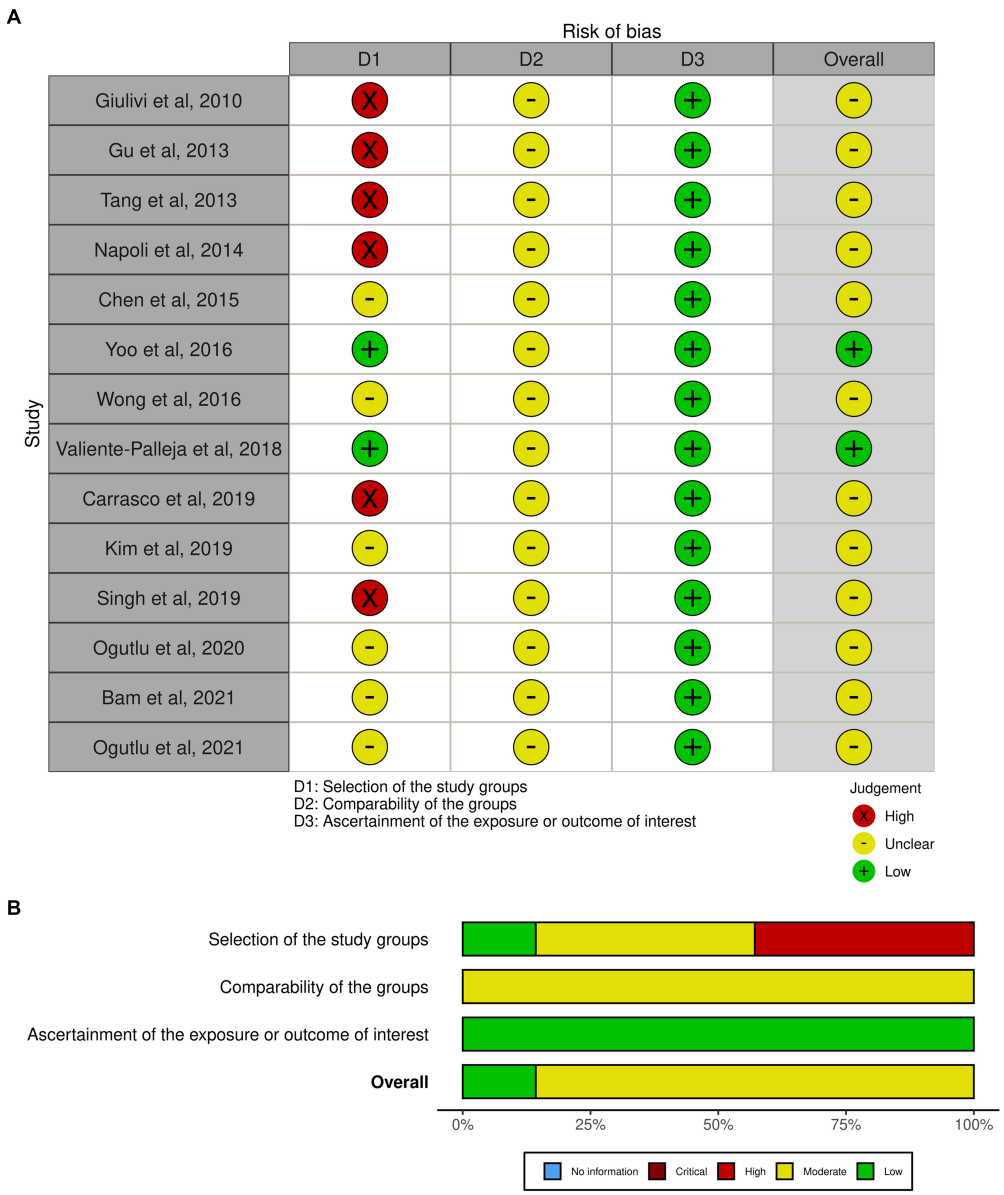
Assessment of the risk of bias. **(A)** Traffic light plot, **(B)** Summary plot.

The risk of bias assessment in this meta-analysis is presented in graphical format as a traffic light plot (Figure 10A) and a summary plot (Figure 10B). According to the summary plot, about 20% of the studies had low risk of bias while the remining 80% had a moderate risk of bias. High risk of bias was observed in about 40% of the studies and was mainly due to small sample size.

## 4. Discussion

Mitochondria play a key role in energy production and cellular function. They are the only organelles that have their own DNA (mtDNA), which presents in multiple copies per cell. The mtDNA encodes only 37 genes, and 13 proteins translated by these genes are subunits of the electron transport chain (ETC), involved in the oxidative phosphorylation of ATP, providing the body with a steady stream of energy (14, 15). The mtDNA is a direct target of ROS attack due to its *proximity to* ETC, *the source of oxidants*, lack of histones protective proteins and DNA repair capacity. Oxidative stress may ultimately lead to mutations or copy number alteration that could impair the mitochondrial function, triggering a vicious cycle of ROS generation and mtDNA oxidative damage (20–22). Many factors causing mitochondrial dysfunction are directly associated with a wide range of pathological conditions. Among these factors, quantitative changes including high and low mtDNA-cn have been frequently observed in human diseases such as cancer, neurodegenerative diseases and psychiatry conditions (23–25). One of the proposed mechanisms leading to high mtDNA-cn is augmented oxidative stress. It has been shown that the mtDNA is preferentially clonally amplified to make more mitochondria and mtDNA in response to energy deficient. The increase in mtDNA content has been suggested as the early molecular mechanism in response to oxidative stress in human cells to compensate for damaged mtDNA and defective mitochondria (20, 21). Therefore, high mtDNA-cn can be an indicative biomarker of oxidative stress, which is closely associated with mitochondrial dysfunction (17, 20–22). On the other hand, low mtDNA-cn has been linked with poor oxidative capacity, which leads to abnormal oxidative phosphorylation and defective cellular energy production (20–22).

In previous studies, both increased and decreased mtDNA-cn in blood and other tissues have been linked to neurodevelopmental disorders, suggesting a diverse and complicated role of altered mtDNA-cn in these disorders. In this study, we performed a meta-analysis to summarize the current evidence and to provide more conclusive results regarding the relationship between mtDNA-cn and ASD/ADHD. We first evaluated the relationship between mtDNA-cn and ASD/ADHD in an overall meta-analysis. The results showed a significant relationship between higher mtDNA-cn and ASD/ADHD. Although there was a large heterogeneity across the studies, further evaluation showed that the data did not significantly influence the pooled results. The variations in the range of mtDNA-cn could be explained by many factors. While real time qPCR is an accurate and reliable method for mtDNA-cn evaluation, quantification can be affected by the methodological variability in the process. For instance, the techniques used for total DNA extraction and different sets of primers used to amplify the mtDNA genes may affect the range of mtDNA-cn (52). Noticeably, some studies used multiple primer sets for different mtDNA genes (26–28, 37–41) and this can impact the precision of DNA quantification. However, mtDNA-cn could be determined using several mtDNA genes and the variations in primer selection should not affect the outcome of the meta-analysis. The wide variations of mtDNA-cn ratios between studies may be also due to the sample used for DNA extraction such as blood (26–28, 30, 31, 37, 39–41, 43), brain tissues (29, 38), and oral samples (42, 44). Even with the use of blood sample, variations in mtDNA-cn quantification may occur. For instance, platelet contamination during extraction of DNA from peripheral blood mononuclear cells (PBMCs) may lead to inaccurate estimation of mtDNA-cn (53).

The mtDNA-cn varies widely among tissues and can adapt to the energy demand and physiological condition of each individual (16, 17), we evaluated the relationship between mtDNA-cn and ASD/ADHD in stratified analysis by tissue types to reflect the biological differences between mtDNA-cn in the blood vs. non-blood tissues. The analysis of blood-based mtDNA revealed no significant association between mtDNA-cn in blood samples of patients compared to controls. Whereas, a significant relationship between higher mtDNA-cn was observed in non-blood samples of patients compared to controls. However, it is difficult to make a final conclusion on the observed increase in mtDNA-cn in non-blood samples due to the small number of studies conducted on brain and oral tissues.

In the current meta-analysis, blood was the most common tissue examined for mtDNA-cn changes in ASD and ADHD. To reduce the uncertainty induced by blood compositions as confounding factors, further analysis was performed by stratifying studies based on peripheral blood samples only. No significant association was found between higher mtDNA-cn in peripheral blood samples of ASD/ADHD patients compared to controls.

Studies have shown that changes in mtDNA-cn may be influenced by the age of individuals (18, 19). Thus further stratified analyses were conducted on studies reported differences in the mtDNA-cn in blood and non-blood tissues of patients with ASD/ADHD and controls within a range age between 2 and 13 years old. We found no significant difference in mtDNA-cn in blood samples between patients and controls, whereas a significant increase in mtDNA-cn was observed in other non-blood samples between patients and controls.

Blood-derived mtDNA-cn has been proposed as a potential biomarker for many diseases (23–25). However, different tissues have different mtDNA content (16, 17), and it remains unclear how blood mtDNA-cn can reflect disease status in other tissues. In some studies, blood mtDNA-cn was found to be associated with gene expression in other human tissues (54). Conversely, changes in mtDNA-cn in tissue samples were not reflected in blood samples (55). The mtDNA can be released by mitochondrion into the cytosol or bloodstream. The release of mtDNA into the cytosol involves DNA sensing receptors that trigger innate immune signaling (56, 57). Whereas the release of mtDNA into the bloodstream as cell-free mtDNA reflects physiological status (58). It has been shown that under psychological stress, cell-free mtDNA can be produced even in the absence of injury (59, 60), implying that the level and the biological role of cell-free mtDNA differ from tissue mtDNA-cn (61). Moreover, blood mtDNA-cn does not reflect tissue mtDNA-cn or capacity of mitochondrial energy production in other tissues (61).

Variations in mtDNA-cn measured in blood can be caused by differences in blood-cell compositions due to different mtDNA content in different blood-cell types. For instance, no consistent correlation was found between mtDNA-cn in blood leukocytes and cell-free mtDNA in plasma (62). In fact, blood composition is a strong confounder of blood-based mtDNA-cn measurement. Determinant factors of blood-derived mtDNA-cn include cellular heterogenicity of leukocytes and platelet abundance, which can change with physiological and pathological conditions (61). Leukocyte and platelet counts are therefore important sources of variation in whole blood mtDNA-cn measurement (63). Particularly, with the objective of measuring mtDNA-cn in blood to develop a biomarker, precise adjustment for the abundance of the subtypes of blood cells is important to rule out the potential confounders and to improve the specificity of mtDNA-cn as a stable biomarker. More studies are required related to quantification of mtDNA-cn in homogenous or well-defined cell populations to mitigate the possibility of confounding and to elucidate the influence of blood-cell type variations on mtDNA-cn. It is worth noting that nuclear genes involved in mtDNA replication and/or maintenance as well as common genetic variants play important roles in regulating the mtDNA-cn (64, 65). Therefore, the mtDNA-cn is suggested as a complex biomarker reflecting specific mitochondrial function, mostly related to mtDNA regulation which is also under nuclear genetic control (65, 66).

ASD and ADHD are the two most common neurodevelopmental disorders observed in childhood. These disorders affect the brain function and neurological development, causing impairments in cognition, communication, behavior, and motor functioning (1, 2). The brain is a highly energy-consuming organ and approximately 96% of this energy is used by neurons (67). In the developing and mature brain, mitochondria are centre for several pathways crucial for neural development, survival, activity, and connectivity (67). In neurons, mitochondria are also involved in calcium buffering and generation of ROS (68). Therefore, high-energy demand tissues such as neurons are often the most strongly affected by mitochondrial dysfunction.

Neurons are extremely sensitive to free radicals (69, 70). Increased oxidative stress and disrupted intracellular redox status, which contribute to mitochondrial neuronal dysfunction have been previously reported in ASD (71, 72) and ADHD (13). In addition, there is a direct links between increased mtDNA-cn and enhanced oxidative stress in ASD patients (37, 39, 42).

Impaired mitochondrial function can alter brain energy metabolism and cause neurological disorders. The increase in mtDNA-cn may be a compensatory adaptive response to overcome oxidatively defective mitochondria in order to correct for energy deficit (20, 21). Considering these observations, elevated mtDNA-cn in blood and or tissues of patients with ASD and ADHD may be a consequence of dysfunctional state in mitochondria.

Mitochondrial dysfunction can be classified as either primary or secondary defects. Primary mitochondrial dysfunction are caused by defects in mtDNA and/or nuclear DNA (nDNA) genes directly involved in mitochondrial function and ATP producing (73). Secondary mitochondrial dysfunction on the other hand are due to other metabolic and genetic abnormalities or environmental factors that impair the ability of mitochondria to produce ATP (73). The pathophysiology of mitochondrial dysfunction in ASD is very complex and can be caused by defects in mtDNA and/or nDNA genes associated with mitochondrial function as well as other mitochondrial aberrations. A meta-analysis conducted by Rossignol and Frye (74) showed a higher prevalence of mtDNA deletions in patients with ASD than in the general population. Their study also suggested that primary mitochondrial disease may occur in a subgroup of patients with ASD (74). In addition, children with mitochondrial disease or abnormal mitochondrial function may be more vulnerable to external factors causing regression in developmental milestones in ASD including language, motor skills, eye contact, play skills, social interaction and receptive skills (74). Whereas abnormalities in several biochemical markers of mitochondrial dysfunction such as elevated lactate and lactate-to-pyruvate ratio and/or toxic environmental exposures can result in secondary mitochondrial dysfunction in children with ASD (74). Another study by Varga et al. (75) showed that mtDNA alterations including mtDNA deletions are more common in patients with ASD than in control individuals, and coexist either with alterations in nuclear genes associated with ASD and genes essential for mtDNA maintenance or with environmental factors. The high prevalence of mtDNA deletions in ASD patients was suggested as a secondary effect (75). Similarly, mitochondrial dysfunction has been suggested as a crucial mechanism underlying the pathophysiology of ADHD. As such, several mtDNA defects including mtDNA mutations/polymorphisms and abnormal mitochondrial-associated protein biomarkers have been described in patients with ADHD (76). Moreover, mitochondrial defect in cybrid-neurons can significantly alter the serotonergic neurotransmitter function, which contributes to ADHD pathology and/or phenotypes (13). Given that ASD individuals with mitochondrial defects exhibit symptoms of ADHD (8, 9), mitochondrial dysfunction is probably a central, *common* themes in ASD and ADHD. However, the exact genetic etiology of mitochondrial dysfunction in the pathophysiology of these *disorders* remains uncertain.

ASD and ADHD share some clinical features (6), which may be due to shared genetic factors and other influences (8, 9). To further understand the contribution of mitochondrial dysfunction in these disorders, additional stratified analysis was conducted on studies reported differences in mtDNA-cn in the blood of aged-matched patients with ASD or ADHD and controls. No significant difference in mtDNA-cn was observed between ASD patients and controls, while a significant increase in mtDNA-cn was found in ADHD patients than in controls. It should be noted that the blood samples used to measure mtDNA-cn in ASD were peripheral blood, leukocytes, and PBMCs as compared to only peripheral blood samples in ADHD studies. This further supports the earlier observations that blood-cell compositions are important confounders of blood-based mtDNA-cn measurement (61–63).

Although brain tissues are considered as the standard target tissue in studying neurodevelopmental disorder and other brain disorders, blood is minimally invasive for mtDNA-cn measurement. Nevertheless, blood collection may cause patient’s discomfort and requires a standard level of training. Saliva is less invasive, accessible biofluid that can be collected by patients following simple instructions. Salivary mtDNA is also independent of the number of cells in saliva, thus it is not confounded by cellular content (77). Recently, a number of studies have highlighted the usefulness of salivary mtDNA-cn as a possible biomarker to monitor patients with head and neck cancers (78, 79), and to study dynamic neuroendocrine changes (77). Furthermore, saliva has been used to study cell-free mtDNA-cn in children and adults with stress and trauma (80). These observations suggest several advantages of salivary mtDNA-cn over blood and other invasive tissues.

Taken together, findings from *studies* investigating the pathogenesis of ASD and ADHD have suggested an important role of mtDNA-cn in these conditions, making mitochondria a potential target for effective *therapeutic* strategies (68). Improving the biological specificity of tissues used in mtDNA-cn quantification is important for the utility of mtDNA-cn as a promising biomarker in neurodevelopmental disorder.

Certain limitations in our study should be considered. First, we only included a small number of studies, only two publication measured mtDNA-cn in brain tissues and two publications measured mtDNA-cn in oral samples out of 14 included studies, and future analyses are warranted in larger studies. Second, lack of sufficient information about behavioral and clinicopathological characteristics of patients limited our further evaluation of these factors with mtDNA-cn. In addition, lack of randomized controlled trials reduced the reliability of the pooled outcome.

To our knowledge, this is the first meta-analysis to evaluate the relationship between mtDNA-cn and ASD/ADHD. Our overall analysis of mtDNA-cn in all blood samples and non-blood samples (brain tissues and oral samples) suggest elevated mtDNA-cn in patients than in controls, further emphasizing the implication of mitochondrial dysfunction in neurodevelopmental disorders. However, our results indicate that the mtDNA-cn in blood is not reflected in other tissues in ASD/ADHD, and the true relationship of blood derived mtDNA-cn remains to be elucidate in future studies. Our results also highlight the importance blood-cell compositions as confounder of blood-based mtDNA-cn measurement and the advantages of salivary mtDNA-cn over blood and other invasive tests. Finally, the potential of mtDNA-cn as a biomarker for mitochondrial malfunction in neurodevelopmental disorders deserves further investigations.

## Data availability statement

The original contributions presented in the study are included in the article/supplementary material, further inquiries can be directed to the corresponding author.

## Author contributions

GA-K: conceptualization, methodology, study design, retrieving, processing and analysing data, writing the original draft, and reviewing and editing the manuscript. HJ: conceptualization, methodology, study design, retrieving, processing and analysing data, and editing and reviewing the manuscript. YA, MA and MH: supporting processing and analysing data, and editing and reviewing the manuscript. All authors contributed to the article and approved the submitted version.

## Conflict of interest

The authors declare that the research was conducted in the absence of any commercial or financial relationships that could be construed as a potential conflict of interest.

## Publisher’s note

All claims expressed in this article are solely those of the authors and do not necessarily represent those of their affiliated organizations, or those of the publisher, the editors and the reviewers. Any product that may be evaluated in this article, or claim that may be made by its manufacturer, is not guaranteed or endorsed by the publisher.
